# Lived body and the Other’s gaze: a phenomenological perspective on feeding and eating disorders

**DOI:** 10.1007/s40519-020-01103-2

**Published:** 2021-02-05

**Authors:** Milena Mancini, Cecilia Maria Esposito

**Affiliations:** 1 Department of Psychological, Humanistic and Territorial Sciences, University “G. D’Annunzio”, Via Dei Vestini 31, 66013 Chieti, IT Italy; 2 Department of Pathophysiology and Transplantation, University of Milan, Milan, Italy

**Keywords:** Eating disorders, Lived body, Optical-coenaesthetic disproportion, Phenomenology, Psychotherapy

## Abstract

According to the phenomenological perspective, the lived body disorder is a core feature of feeding and eating disorders (FEDs). Persons with FEDs experience their own body first of all as an object looked by another person, rather than coenaesthetically or from a first-person perspective. In particular, the main features of this disorder are: alienation from the own body and from the own emotions, disgust for it, shame, and an exaggerated preoccupation for the way in which one appears to the others. Phenomenological research has recently highlighted that the gaze of the Other plays an important role. Because persons with FEDs cannot have an experience of their own body from within or coenesthetically, they need to apprehend their own body from outside through the gaze of the Other. This way of apprehending one’s own body when it is looked by another person is called by Sartre the ‘lived body-for-others’. Normally, the constitution of one’s own body, and consequently of one’s own Self and identity depends on the dialectic integration between the first-person apprehension of one’s body (lived body) that it is based on coenaesthesia, and the third-person one, that it is based on the sense of sight (lived-body-for-others). When the dialectic is unbalanced toward the pole of the lived-body-for-others, experienced from without, the symptom occurs. Starting from these clinical observations, the so-called Optical-Coenaesthetic Disproportion model has been developed. In this paper, we describe this model, its philosophical and clinical foundations, and finally its clinical implication and its relationship with other disciplines, i.e., neurosciences. Level of evidence: V.

## Introduction

Feeding and eating disorders (FEDs) are a series of disorders extremely frequent in our time, on the bases of which have been described disturbances in embodiment and in resulting identity-shaping process [1, 2]. Although classical nosography mainly emphasizes the behavioural aspect of these disorders, clinical observation shows that FEDs cannot be reduced only to behavioural disturbances, but they contain deeper experiential alterations [2]. In fact, common clinical features are the difficulty in perceiving emotions, the anomalies of body experience, the search for an ideal body image, as well as the instability of the identity construct [3, 4]. Starting from these observations, clinical phenomenology has not just the prerogative to look only at behaviours, but also to identify the experience that underlies them. In fact, it does not appear as a pure clinical description, but provide the conditions for establishing pathogenetic models. What unifies the various clinical manifestations of these disorders is a common psychopathological nucleus—an abnormal experience of corporeality [4–8]. Lacking the balance between body subject and body object and between body experienced from a first-person perspective and body experienced from a third-person perspective, persons affected by FEDs find themselves suspended in an unresolved dialectic that generates the symptom. In fact, a specific kind of lived body disorder demonstrated both from a clinical and neuroscientific point of view, appears the central pheno-phenotype on which these disorders are built [9].

Specifically, to understand how these experiences of one’s own body hold together, the Optical-Coenaesthetic Disproportion Model has been developed [10]. According to this hypothesis, FEDs derive from an anomalous coenaesthetic apprehension of one's body, which is perceived fragmentary and shifting, constituting an unstable experience of the Self. This original alteration results in the compensatory hypertrophy of the optical apprehension of the Self, which is conveyed by the gaze of the other, and reduces subjectivity to the body object. The gaze of the other, therefore, performs the function of a visual prosthesis that allows these subjects to feel their own body, whose experience from within is lacking [11].

Understanding how persons affected by FEDs experience their body opens the possibility of intervening therapeutically not only on behaviours, but also on those experiences that give rise to behaviours themselves [12].

### Dimensions of corporeality in FEDs

Phenomenology, with the contribution of philosophers as well as psychopathologists and clinicians, has allowed to deepen the theme of the corporeality and the experience of the person in this regard, opening fruitful perspectives from a clinical point of view [13, 14].

Our body lies in a situation of constitutive ambiguity [15], since the experience that we have is based on the dialectic between body subject and body object and between subjectivation and reification. On one hand, it is experienced from within, as body subject, which corresponds to the body that we feel as ours, which we experience in first-person perspective, to the *body-I-am*. This body is the starting point and the inevitable point of view of all our experiences in the world, from which every process of subjectivation begins. It represents a body that we feel ours, in which we identify ourselves to the point of experiencing it as a simple physical extension of our will. The apprehension of its needs occurs on a pre-reflective level and expresses the spontaneity of our being-in-the-world [16].

On the other hand, our body is also experienced as a body object. In this sense, it is configured as something independent of our will, which can be objectified through biomedical measurements or—as we will see specifically in this paper—through the reification imposed by the gaze of the other [17]. This second experience of the body is often felt as constricting, resistant to any attempt to modify it, not fully corresponding to what we wish of it. It is the *body-I-have,* lived in the third-person perspective, a burden that weighs us down on materiality [18]. The body object corresponds to an experience of corporeality which shows us it is contingency: a body that gets sick or tired, that weighs with respect to a will that would always want it ready and quick. The object-like experience of our body occurs in particular in the encounter with the other, which gives us an image of us crystallized in our body: the gaze of the other becomes something that limits us to our body object, forcing us to deal with the reification that this entails [17].

Husserl had already described the difference between *Leib* (lived body) and *Körper* (body object) [15], but a more complete development of these concepts will take place in French phenomenology, in particular in the works of Merleau-Ponty and Sartre. These two philosophers, although coming from the common assumption of Husserl’s perspective, have followed different paths, each one developing a particular aspect of his own thinking.

Merleau-Ponty is particularly responsible for the elucidation of the theme of the *flesh* (*chair*). The *flesh* corresponds to our body perceived from within, to that amalgam of experience that finds its primitive organization in the coenaesthetic apprehension of myself [16, 19]. Coenaesthesia is the global experience in which all the single bodily sensations are synthesised, the crossroads of all interoceptive sensibility on which self-consciousness is grounded, including the feeling of existing, of being a self, and of being separated from the external world [4, 10, 11, 19]. In FEDs, as we will see, the *flesh* is experienced as unstable and shifting. Being the *flesh* the foundation of our being-in-the-world, not feeling it or feeling it as evanescent means not belonging to the world. For this, it becomes necessary to find a new anchor in the world.

According to the Optical-Coenaesthetic Disproportion model, the hypertrophy of the optical experience of one's own body responds to the lack of the coenaesthetic experience. Sartre introduces a third experiential dimension of the body, in addition to the body subject and the body object that is the lived-body-for-others. The body is conceived as an object-to-be-seen, vulnerable to the gaze of the other, as a mark of what is independent of our will but to which we are nevertheless subdued [20]. The gaze of the other, in Sartre’s perspective, is alienating and reifying, because it reduces me only to my visible body. However, the primitive and inevitable alienation conveyed by the gaze of the other is at the same time the only ground from which subjectivation can begin—according to the famous maxim with which "the important thing is not what has been done to us, but what we ourselves do with what has been done to us" [17]. The identity-making process, starting from the lived-body-for-others, then resolves itself into the balanced dialectic between body subject and body object.

The way I feel when I am seen by others (lived-body-for-others) may or may not correspond to the way that I feel my body in the first-person perspective (body subject) and in the third-person perspective (body object). The integration between these three ways to experience own body allows for a stable identity.

### The optical-coenaesthetic disproportion hypothesis

#### Clinical background

The Optical-Coenaesthetic Disproportion hypothesis of feeding and eating disorders (FEDs) has its roots in the dissatisfaction with standard definition and classification of this syndrome, based on the focus on behavioural or observable anomalies (e.g., restriction of energy intake, low bodily weight, and behaviour which interferes with weight gain) and biased by value-laden concepts (e.g., undue influence of body shape, and lack of recognition of the seriousness of the current low body weight). In addition, one of the main issues in the classic nosography of FEDs is that patients move between various diagnostic categories across time (diagnostic crossover) [21, 22]. Longitudinal studies indicate that large proportion of patients migrate among diagnoses over time [21–23] without a substantial change in basic psychopathological features [24] suggesting the existence of a common psychopathological core [3, 4, 7, 14, 25–27].

From a phenomenological perspective, the abnormal experiences that characterize the persons with FED (e.g., weight and shape concerns) are the result of a more profound disturbance which concern to the way these persons experience their own body (embodiment) and shape their personal identity. [2–4, 7, 14, 25, 28]. In line with this perspective, the assessment of persons with FEDs should include the way of experiencing one’s own body and lived corporeality enriching and going beyond the behavioural evaluation required for DSM diagnosis. Clinicians should consider the impact of this dimension on other aspects of the patient’s life, namely the role of the body both in the intersubjective interactions and in the process of shaping of the personal identity, as well as in other dimensions associated with FED such as the way of experiencing the space and the time [2, 4, 14, 25, 26]. Indeed, in literature, behaviours and cognitive distortion that characterize FED can be understood in the light of spatial metamorphosis that is deeply associated with the disorder of corporeality [25] as well as the experience of time that these patients try to achieve by controlling eating and weight [29].

The focus on the experiential dimension of FEDs brought in the foreground clinical features related to bodily experience and to the process of identity formation in these persons through devising an assessment tool for empirical research called IDEA (IDentity and EAting disorders) [7]. First, this questionnaire allowed to collect clinical data in large populations of persons affected by FEDs, or vulnerable to it, as well as in the general population or in patients affected by cognate disorders like obese bariatric patients. In a subsequent step, the data obtained through the IDEA questionnaire became the basis to identify and describe a specific pheno-phenotype [14, 26, 31, 32] that expresses a gradient of vulnerability to FEDs along a continuum, rising from high-risk nonclinical subjects toward the clinical population of eating disorder patients and including obese patients [33, 34]. The pheno-phenotype tracked down is a specific type of disorder of embodiment, in particular the troubling of the *coenaesthesia apprehension*, that is the experience of my body from within, in which interoceptive and proprioceptive sensibility conveys [11–35].

The Optical-Coenaesthetic Disproportion hypothesis has been developed from these data.

#### Description and details of optical-coenaesthetic disproportion hypothesis

The Optical-Coenaesthetic Disproportion hypothesis argues that in persons with FEDs, the coenaesthetic apprehension of oneself is troubled, and as a compensation to it, these persons experience their own body as an object that is looked at by the other [9].

Each of us, under normal conditions, experiences his own body through the combination of the way we feel ourselves from a first-person perspective and the way we experience ourselves through other sense modalities, in particular the sight. Therefore, bodily experience is the result of a dialectical or proportion between two modalities: coenesthetic and visual.

Therefore, the constitution of the subject develops in the dialectics between these two phenomena, both necessary but partial, which, however, in mutual interaction manage to provide the foundations for the development of the identity of the subject. In fact, our identity-making dialectics always starts, necessarily, from the basis that our experience of the body provides us, and only later it can distance itself from its complete adherence to materiality and criticize it [17, 18, 36]. The constitution of these two dialectical poles, body subject and body object, reflects two different ways of experiencing one’s own body: on one hand, the coenaesthetic apprehension, which corresponds to one's own body experienced from within, on the other, the optical perception, which corresponds to the body seen from the other’s vantage [10, 11]. These two ways of experiencing our body, both valid and necessary, must however be in balance with each other to allow the rise of a healthy subject. Otherwise, when a pole becomes predominant, disproportion and therefore psychopathology are encountered [14].

In persons affected by FEDs this dialectical mode is disturbed and their possibility to feel themselves is weakened. Facing a sense of unfamiliarity with one’s own flesh, they compensate their disturbed coenesthesia, experiencing their own body as an object being looked at by another, rather than coenesthetically or from a first-person perspective. The peculiar mode to experience their own body in these patients is “*Videor ergo sum*” (“I am seen therefore I am”) [37].

The way they feel looked at by the other is the principal mode to feel themselves and define their identity [2, 14, 26]. Their body is principally given to them as an object “to be seen.” It is a body-for-the-other, that is a body exposed and subjected to the other’s gaze and thus reduced to its appearance.

Here, the other’s look is a kind of visual prosthesis that help them to feel their own body. Feeling one’s body as an object being looked at by another has two effects: (a) it makes FEDs people feel embarrassment and repulsion for their own body, (b) but it also helps them recover a sense of selfhood, “unity,” and “condensation” [14, 26, 31].

The characteristics of the body object then become the only ones that can guarantee me as a subject, taking over the possibility of defining myself from within. The image of my body, as it appears to the gaze of the other, is no longer one of the elements that form the mosaic of my person, but the main piece of the picture, perhaps even the only one, which defines what I am. In consequence, the frenzy and the fury in trying to make that body-I-have, the visible body, all the more mirror of the body-I-am, of the body-I-feel, are not surprising [12]. The body image therefore becomes a blackboard of self-expression, but always expresses a reified body, an adhesion to the body object that the gaze of the other offers me [9–11, 14, 26]. The vehicle of the identity constitution, instead of the body-I-am, then becomes the measurements of the objective body (BMI, weight, thinness, etc.), as fixed points in a floating world [14, 26] (Table 1a, b).

**Table 1:** **a** Vignette 1: coenaesthetic apprehension troubled. **b** Vignette 2: feeling through the gaze of the other

a
General description
S. S. is a 35-year-old woman who was been affected by eating disorder since the age of 20 Her life-world is characterized by feelings usually experienced as evanescent. Several times S. reports that she is not able of feeling her emotions and she does not know who she is. In her world, what is at stake is the possibility of feeling through one’s body. The experience of not feeling her own body and emotions affects her whole sense of identity. These difficulties were “managed” by S. by continuously measuring her body, by counting the calories ingested and through other compensatory modalities. As if these modalities could help her in feeling herself, in obtaining an identity. This necessity to endlessly construct herself is accompanied by anguish with respect to the inexorable passing of time
Phenomenological file
Emotions: Disgust, shame and anxiety. They are evanescent or extraneous Body: She feels extraneous from her bodily self: “I do not trust what I feel in my body”; “Often, I feel my body as an empty shell”; “I don't feel my body … I want to feel the bones” Time: She experiences time as uncontrollable and sometimes passing too fast. “If I could choose, I would stop time! I promised to myself to lose weight”

b
General description
M. F. is a 24-year-old girl. She alternates moments in which she desires to be seen by other persons to moments when she avoids them. Her possibility to feel herself depends on the gaze of other. Indeed, when M. is in the midst of people, she observes how she is looked at. In this regard, she says: “The way I feel depends on the way I feel looked at by the others”. It is a world full of gazes that save she from feeling her identity as inconsistent
Phenomenological file
Body: Lived body for the other. “Sometimes being looked at by another person gives me a sensation solidity other times I feel excessive and fat. The gaze of other make me feel!” Self: Pornographic conceptualization [52] of the Self that is based on being seen and on the approval others Other: The Other is reduced to its gaze

#### Neurosciences research and optical-coenaesthetic disproportion hypothesis

The Optical-Coenaesthetic Disproportion hypothesis and its underlying clinical data nicely fit with neuroscientific data [9]. Neuroscience research suggests that people with FED have impaired both body image (BI) and bodily self-consciousness (BSC). BI refers to inputs from the body [38] (it encompasses a perceptual, an affective, and a cognitive domain) [11], while BSC is the multisensory integration of the afferents coming from within and from outside the body (exteroceptive inputs) [39]. Neurosciences research shows a dimming of both interoceptive and exteroceptive signals with a predominance of visual signals (exteroceptive) as compared to those coming from inside the body (interoceptive). This dominance of the exteroceptive signals seems to play a fundamental role in abnormal body and self-experiences in people with FED [8, 40–49].

In summary, what is emerged from these studies was a defective constitution of the BSC [9]. On one hand, these empirical data confirm FEDs as disorders of embodiment, and on the other hand, they help to better understand the bodily experiences in FED, going beyond behaviour symptoms. The ALH hypothesis further explains the role of bodily experiences in FED. In particular, it shows how a defect in the integration of the various signals coming from both inside and outside of the body can be explain the symptoms of the FED [46, 50].

Indeed, a compromised integration between the egocentric experience of the body (body-centered) and its allocentric representation (centered on the world/object) does not allow constant updating of the contents of the allocentric representation of the body. The consequence of this impairment is that the subject is locked to the allocentric representation of his body, which primes the processing of any further body-related experience [9].

In conclusion, taken together, all these empirical data highlight an impaired BSC in persons with FEDs, confirming each other in drawing attention to three anomalous features of bodily self-experience: diminished or impaired interoception/coenaesthesia, increased esteroception via visual inputs, and abnormal integration between these two sources of bodily self-experience.

## Psychotherapeutic perspectives

The understanding of FEDs as disorders of the dialectics between body subject and body object, and as the disproportion between the coenaesthetic and the optical perception of one's own body, opens up new therapeutic perspectives. The Optical-Coenaesthetic Disproportion model would in fact allow to focus, in the therapeutic interviews, the theme of the patient's identity-making process and not only his eating-related symptoms. Letting the patient describe the way in which he feels looked at by the other, or how he would like to be looked at, allows us to identify the pathogenetic potential of the gaze of the other. The onset of the symptoms could therefore be related to an interpersonal circumstance in which the patient felt looked at in a certain way. This observation opens up the possibility to deepen those situations in which the experience of the body as an object-to-be-seen comes into play, and consequently the way in which the relationship with the other mediate the identity-making process. The failure of the integration between the body experienced from within and the body experienced from without leads to the collapse of the identity-forming dialectics and contributes to the emergence of the symptom.

The psychotherapeutic relationship, mediating the experience of recognizing the other despite its fragilities and weaknesses, offers the subject a different Other than the one with the reifying gaze that he has always known [12, 31, 51]. The psychotherapist reveals to the subject that there is the possibility of renouncing the alienating effect of the gaze of the other, in favor of an experience of intimacy [14, 26]. Through the use of tact and with the establishment of an effective therapeutic relationship, the blocked identity-making dialectics of these subjects is reactivated. Body subject and body object, instead of being two poles sclerotized in a rigid system, return to interact dialectically with each other. The passage through the therapist’s gaze, a recognizing and non-judgmental gaze, allows the subject to look at his symptom with greater distance, without fully adhering to it, and this is the first step to get rid of it [12].

In this perspective, the phenomenological file becomes a useful tool for looking at the patient's world [13]. The dimensions of subjective experience are organized in the different domains of the phenomenological file (Table 1a, b), which include the experience of time, space, body, other, and self [14, 26]. Consequently, this tool allows to highlight the psychopathological core of the disorders, demonstrated both by clinic and by empirical studies, analyzing how it declines in the various dimensions of personal experience. The implementation of the therapeutic path in this sense appears extremely relevant. The understanding of the dialectics between body subject and body object thus becomes the fundamental pillar of phenomenological-dynamic psychotherapy in patients with FEDs. Even outside of this framework, however, it is a model capable of highlighting therapeutic perspectives that can be addressed within psychotherapies of various orientations, facilitating the understanding of the patient’s being-in-the-world and patient-therapist dialogue. In fact, within a positive relationship dynamic, the subject affected by FEDs can find the engine that allows him to restart the fixed mechanism of his own body experience.

In conclusion, this study shows that, contrary to accounts from mainstream literature, feeding and eating disorders are more than just behavioural anomalies. The hypothesis we described highlights how behavioural anomalies are secondary epiphenomena to a more basic psychopathological core, consisting in disorders of the way persons experience their own body and the way they shape and construct their personal identity (i.e., what it is like to suffer from feeding and eating disorders).

This hypothesis goes beyond mere symptom assessment and, by focusing on anomalies of bodily experience and identity, allows to understand the sufferings of people affected by these disorders, to improve the therapeutic practice by strengthening therapeutic alliance and dialogue, and to promote long-term effects as it is not based only on the elimination of anomalous eating behaviours. Furthermore, this perspective allows a more accurate diagnosis, for example it helps to better distinguish “nuclear” forms of anorexia nervosa from other forms of food intake reduction (e.g., depressive or delusional forms) which differ from the former in terms of specific motivational states. Last but not least, this hypothesis provides a solid epistemological foundation for future research, in particular for neuroscientific research.

### Limits

This is a theoretical paper focusing on the phenomenological understanding of feeding and eating disorders. Its main limitation is that it does not provide new empirical data, yet it is supported by existing clinical evidence taken from the phenomenological perspective.

## Data Availability

Not applicable.
